# Comparison of the diagnostic efficacy of mathematical models in distinguishing ultrasound imaging of breast nodules

**DOI:** 10.1038/s41598-023-42937-x

**Published:** 2023-09-25

**Authors:** Lu Li, Hongyan Deng, Xinhua Ye, Yong Li, Jie Wang

**Affiliations:** 1 Department of Ultrasound, The First Affiliated Hospital, Nanjing Medical University, Nanjing, 210029 China; 2 Institute of Food Safety and Nutrition, Jiangsu Academy of Agricultural Sciences, 50 Zhongling Street, Nanjing, 210014 China; 3 Department of Radiology, The First Affiliated Hospital, Nanjing Medical University, Nanjing, 210029 China

**Keywords:** Biological models, Diseases, Oncology

## Abstract

This study compared the diagnostic efficiency of benign and malignant breast nodules using ultrasonographic characteristics coupled with several machine-learning models, including logistic regression (Logistics), partial least squares discriminant analysis (PLS-DA), linear support vector machine (Linear SVM), linear discriminant analysis (LDA), K-nearest neighbor (KNN), artificial neural network (ANN) and random forest (RF). The clinical information and ultrasonographic characteristics of 926 female patients undergoing breast nodule surgery were collected and their relationships were analyzed using *Pearson*'s correlation. The stepwise regression method was used for variable selection and the Monte Carlo cross-validation method was used to randomly divide these nodule cases into training and prediction sets. Our results showed that six independent variables could be used for building models, including age, background echotexture, shape, calcification, resistance index, and axillary lymph node. In the prediction set, Linear SVM had the highest diagnosis rate of benign nodules (0.881), and Logistics, ANN and LDA had the highest diagnosis rate of malignant nodules (0.910~0.912). The area under the ROC curve (AUC) of Linear SVM was the highest (0.890), followed by ANN (0.883), LDA (0.880), Logistics (0.878), RF (0.874), PLS-DA (0.866), and KNN (0.855), all of which were better than that of individual variances. On the whole, the diagnostic efficacy of Linear SVM was better than other methods.

## Introduction

Breast cancer is one of the most common malignancies in women and poses a significant risk to their health^1,2^. In recent years, the incidence of breast cancer has increased year by year^3^. Early diagnosis, early treatment and tumor biology determine the prognosis of patients^4^. Therefore, how to diagnose breast cancer is very important for patients. Ultrasound is widely recognized as a convenient and safe method for screening breast cancer^5,6^. The ultrasound indicators, especially the shape, direction, edge, internal echo and internal blood flow grading of the nodules, are related to the benign and malignant breast nodules^7^. However, the misdiagnosis rate of benign and malignant breast nodules based on a single ultrasound index is high^8^. Therefore, it is necessary to conduct a comprehensive analysis of multi-index ultrasound characteristics.

The Breast Imaging Reporting and Data System (BI-RADS) lexicon was established to ensure standardization and objectivity in ultrasound diagnosis^9^. However, this grading partially depends on the sonographer's experience^10^. Some benign and malignant breast nodules were misclassified, especially by young physicians. Misdiagnosing these benign breast nodules as malignant may lead to unnecessary biopsies^11^. Many have reported that mathematical models coupling with ultrasound characteristics could serve as a means to automatically discriminate among diseases^12,13^. Logistic regression (Logistics)^14^, support vector machine (SVM)^15^ and artificial neural network (ANN)^16^, partial least squares discriminant analysis (PLS-DA)^17^, linear discriminant analysis (LDA)^18^, K-nearest neighbor (KNN)^19^, and random forest (RF)^20^ are commonly used models for disease diagnosis. Logistics, PLS-DA and LDA belong to linear models, while ANN, KNN and RF belong to non-linear models^21^. SVM could solve linear and non-linear classification problems^15^. Each model possesses its own distinct computational characteristics, however, few studies have compared the diagnostic performances of these diagnostic models. In the present study, we compared the diagnostic effects of ultrasonic imaging combined with different mathematical models, including Logistics, SVM, ANN, PLS-DA, LDA, KNN and RF, in identifying benign and malignant breast nodules.

## Materials and methods

### Patient information

This study was a retrospective study with no adverse effects on patients and was approved by the Institutional Review Committee of the First Affiliated Hospital of Nanjing Medical University. Breast nodules from female patients undergoing ultrasound examination were collected at the First Affiliated Hospital of Nanjing Medical University from June 2018 to December 2022. The inclusion criteria required complete clinical data for patients and confirmation of nodule lesions through pathology. Borderline diseases such as lobulated tumors of grade II were excluded. All images were independently evaluated by two senior physicians with more than a decade of experience. Doctors would convene to discuss and reassess controversial issues such as the grading of nodule features. If a consensus cannot be reached, the controversial images will undergo further review by a third senior doctor and a discussion will be held to reach a final conclusion. A total of 926 breast nodules were included, comprising 388 benign and 538 malignant ones. The cases of benign nodules included fibroadenoma, adenosis, intraductal papilloma, fibrocystic disease of the breast, lobulated tumors of grade I, hyperplasia of glands, and cyst with inflammatory changes. The cases of malignant nodules included mucinous breast cancer, solid papillary carcinoma of breast, invasive breast cancer (invasive lobular carcinoma, invasive ductal carcinoma and mixed invasive carcinoma), and ductal carcinoma in situ.

### Instruments and methods

The birth number, menarche and breast appearance (redness, swelling, and dimples) of patients were recorded. A thorough breast ultrasound was performed using ESAOTE MyLab Twice color Doppler ultrasonic diagnosis instrument with linear array high-frequency probe LA523 and the frequency of 3 ~ 12 MHz probe frequency. The initial preset conditions were as follows: imaging gain at 65%, dynamic range at 10, enhancement at 4, density at 1, depth at 44 mm, persistence at 6, dynamic compression at 3, and transducer resolution set to low (RES-L). In practice, the B-mode image is adjusted to incorporate the target lesion according to patient actual situation to achieve optimal resolution. In the color Doppler ultrasound mode, the sampling frame size was optimized to fully encompass the mass. The color gain was optimized, enabling the detection of low-velocity vascular flow within target lesions with minimal background noise. Ultrasound features included background echotexture, nodular size, shape, margin, internal echo, echo intensity, calcification, alder grade, resistance index and axillary lymph node. These ultrasound features for each nodule were graded according to the BI-RADS lexicon^9^. Background echotexture can be classified as either homogeneous or heterogeneous. Homogeneous background echotexture is defined by the predominant presence of parenchyma displaying a uniform hyperechoic appearance with minimal isoechoic or hypoechoic characteristics and less than 25% fibro glandular tissue^22^. Other background echotextures are defined as heterogeneous type.

Pathological diagnosis of nodules was taken as the dependent variable (Y), and the above ultrasound features and patient’s clinical information were taken as independent variables (X). The assignment of these variables is shown in Table 1.

**Table 1:** Information on the variable assignment.

Factors	Assignment
Age	X1, continuous variable
Birth number	X2, 0 for no birth; 1 for birth with 1 child; and 2 for birth with more than two children
Menarche	X3, 1 for 11-12 years old; 2 for 12-14 years old; and 3 for ≥14 years old
Breast appearance	X4, 0 for normal; 1 for heat, redness, swelling or pain; and 2 for dimpling sign
Background echotexture	X5, 0 for homogeneous; and 1 for heterogeneous
Nodular size	X6, 0 for<1 cm; 1 for≥1 cm&<3 cm; and 2 for≥3 cm
Shape	X7, 0 for regular; and 1 for irregular
Margin	X8, 0 for smooth or clear; 1 for speculation or ill-defined
Internal echo	X9, 0 for anecho; 1 for Isoecho, hyperecho or mild hypoecho; and 2 for marked hypoecho
Echo intensity	X10, 0 for hyper echo& isoecho; and 1 for hypoecho
Calcification	X11, 0 for none; 1 for macrocalcification; and 2 for punctate calcifications or mixed calcification
Alder grade	X12, 0 for Grade 0; 1 for Grade I; 2 for Grade II; and 3 for Grade III
Resistance index	X13, continuous variable
Axillary lymph node	X14, 0 for negative; and 1 for suspicious
Pathological type	Y, 0 for benign; and 1 for malignant

### Statistical analysis

The relationship between ultrasonographic characteristics and clinical information (age, birth number, menarche, breast appearance and pathological type) was analyzed using *Pearson*'s correlation, with P<0.05 indicating a significant correlation. Principal component analysis (PCA) was adopted to analyze the difference between nodule features in benign and malignant groups. Before establishing the models, the relevant variables were selected via the stepwise regression method, where the variable with a P-value < 0.05 was considered to have a significant relationship with the dependent variable^23^. In data analysis, 90% and 10% of 926 nodule cases were randomly divided into training and prediction sets, respectively, using the Monte Carlo cross-validation method^24,25^. In order to mitigate errors arising from a single calculation, Monte Carlo simulation was performed 100 times. The average diagnostic rate of each model for benign and malignant breast nodules in the training and prediction sets over 100 computations was separately computed. The diagnostic efficacy among models was compared by using the area under the receiver operating characteristic curves (ROC) in the prediction set. All the programs of these models were performed using MATLAB software. *t*-test was used to analyze the difference in diagnostic effectiveness among different models, with P<0.05 indicating a significant difference.

### Ethics statement

This study was approved by the Institutional Review Committee of the First Affiliated Hospital of Nanjing Medical University (Approval number: 2022-SR-048) and patient informed consent was waived. This study retrospectively analyzed the ultrasonographic image of the patient's previous examination, which posed no potential risk or harm to the patient. The study was conducted with strict adherence to the Declaration of Helsinki. No patient privacy data was included in the data collection, and the data remained strictly confidential throughout the collection process.

## Result

### Breast nodule features

**Figure 1:**
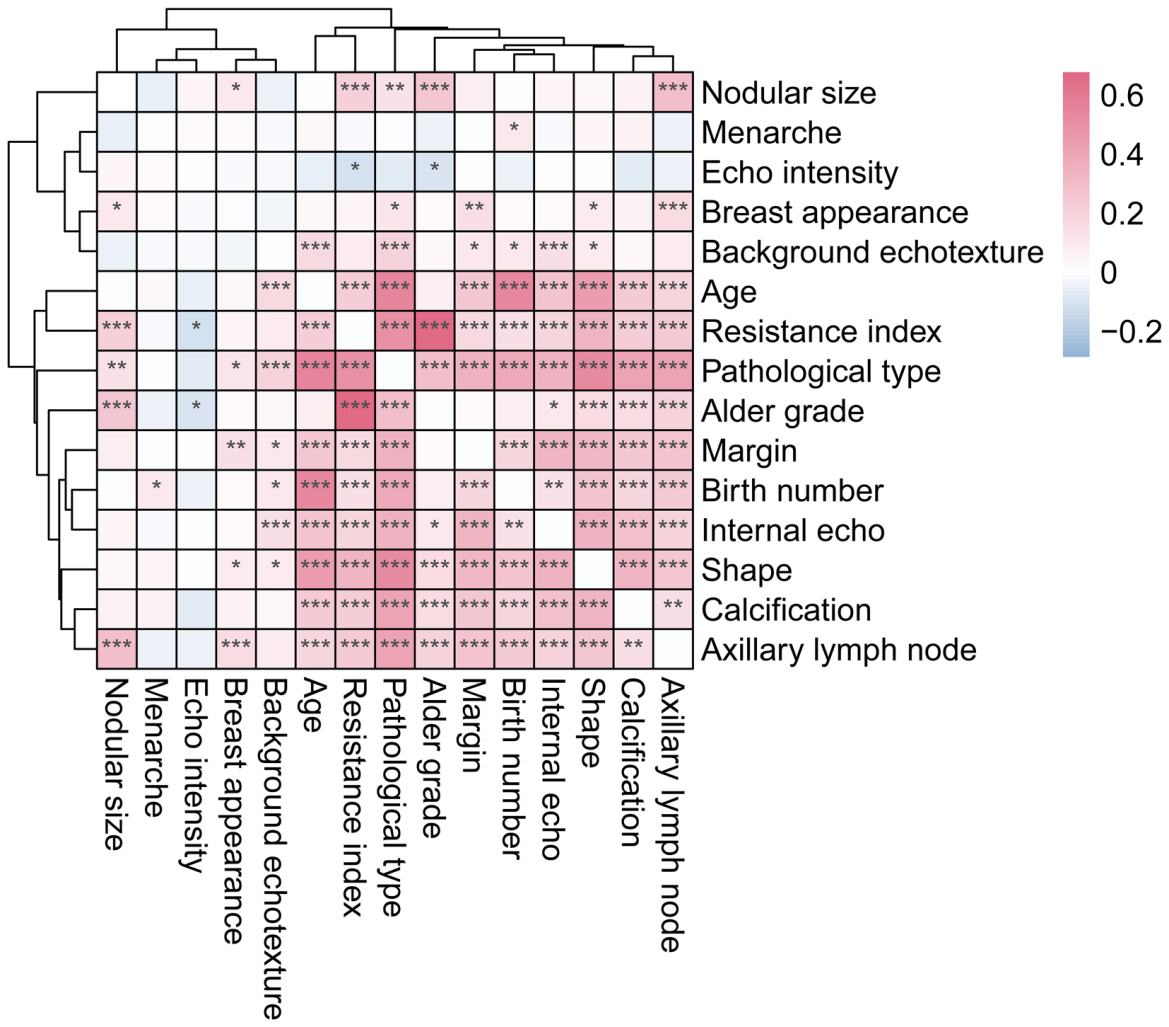
Correlation analysis of ultrasonographic characteristics and clinical information, where *P value < 0.05; **P value < 0.01; ***P value < 0.001.

**Figure 2:**
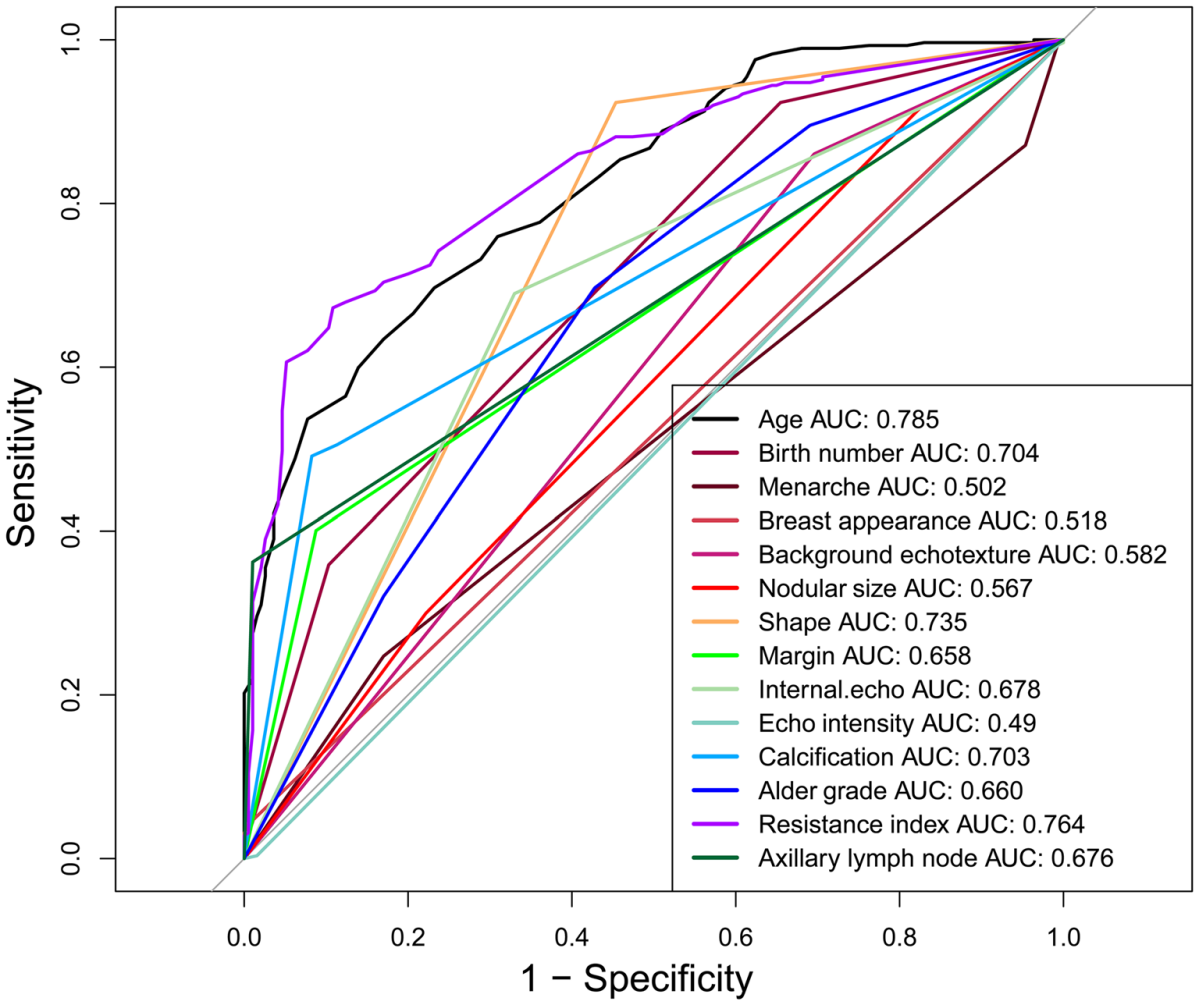
Area under the ROC curve (AUC) of individual variances using Logistics.

A total of 926 breast nodules were collected, including 388 benign nodules (41.9%) and 538 malignant nodules (58.1%). Each breast nodule included 10 ultrasound characteristics and 5 clinical information. Via *Pearson*’s correlation analysis, benign and malignant breast nodules were significantly correlated with patient’s age (r = 0.548, P < 0.05), shape (r = 0.520, P < 0.05), resistance index (r = 0.491, P < 0.05) calcification (r = 0.419, P < 0.05), axillary lymph node (r = 0.414, P < 0.05), birth number (r = 0.389, P < 0.05), internal echo (r = 0.348, P < 0.05), margin (r = 0.346, P < 0.05), alder grade (r = 0.289, P < 0.05), background echotexture (r = 0.200, P < 0.05) and nodule size (r = 0.137, P < 0.05) (Fig. 1). Logistics based on individual ultrasonographic characteristics showed that resistance index has the highest the area under the ROC curve (AUC = 0.764), followed by shape (AUC = 0.735), calcification (AUC = 0.703), axillary lymph node (AUC = 0.676) and alder grade (AUC = 0.660) (Fig. 2). Besides, many correlations were found among ultrasonographic characteristics and clinical information. For example, alder grade was significantly positively correlated with nodular size, shape, calcification, resistance index, and axillary lymph node (P < 0.01). Breast appearance was significantly correlated with margin and axillary lymph node (P < 0.01).

### Principal component analysis and variable selection

**Figure 3:**
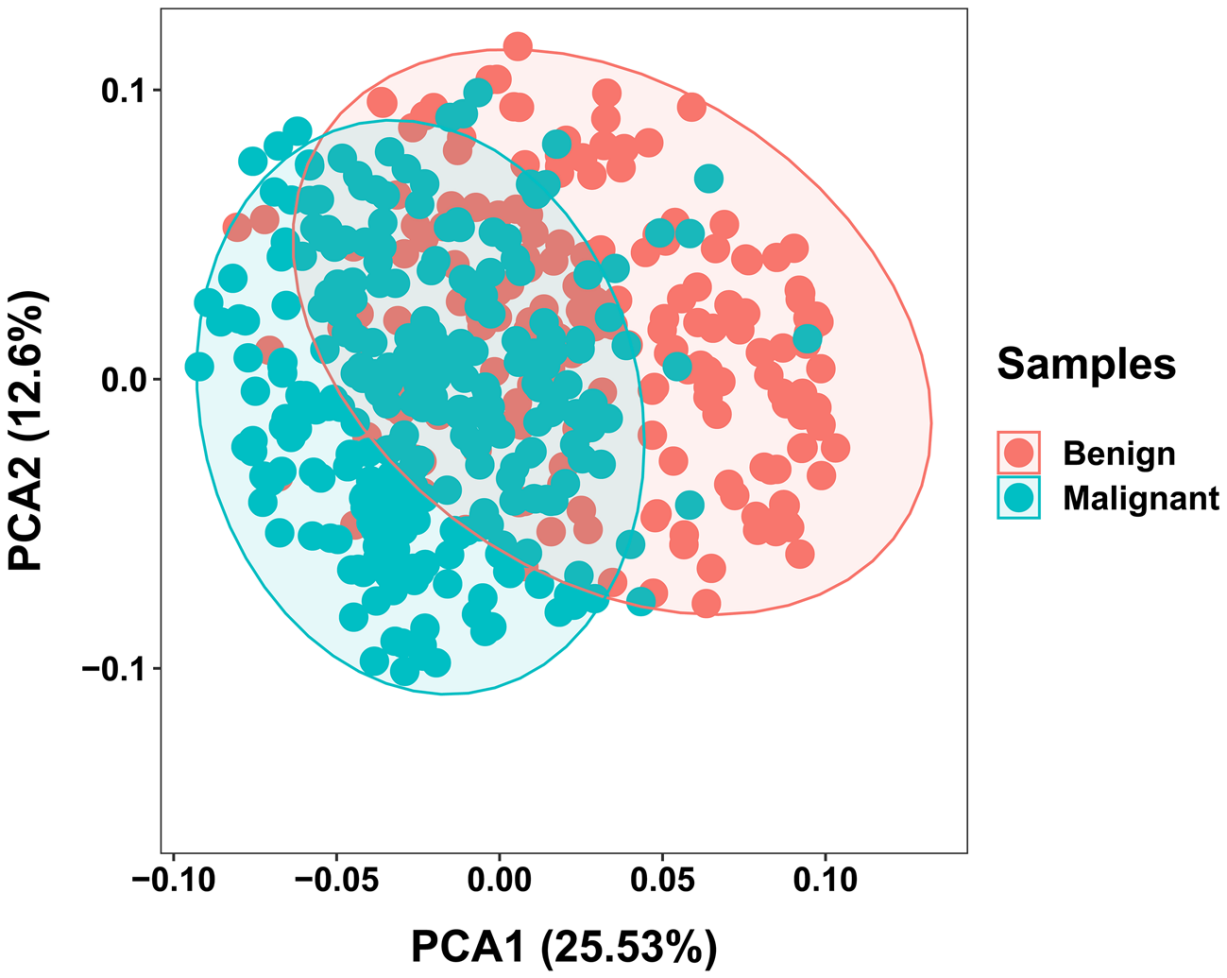
PCA analysis of ultrasonographic characteristics in benign and malignant breast nodules.

**Figure 4:**
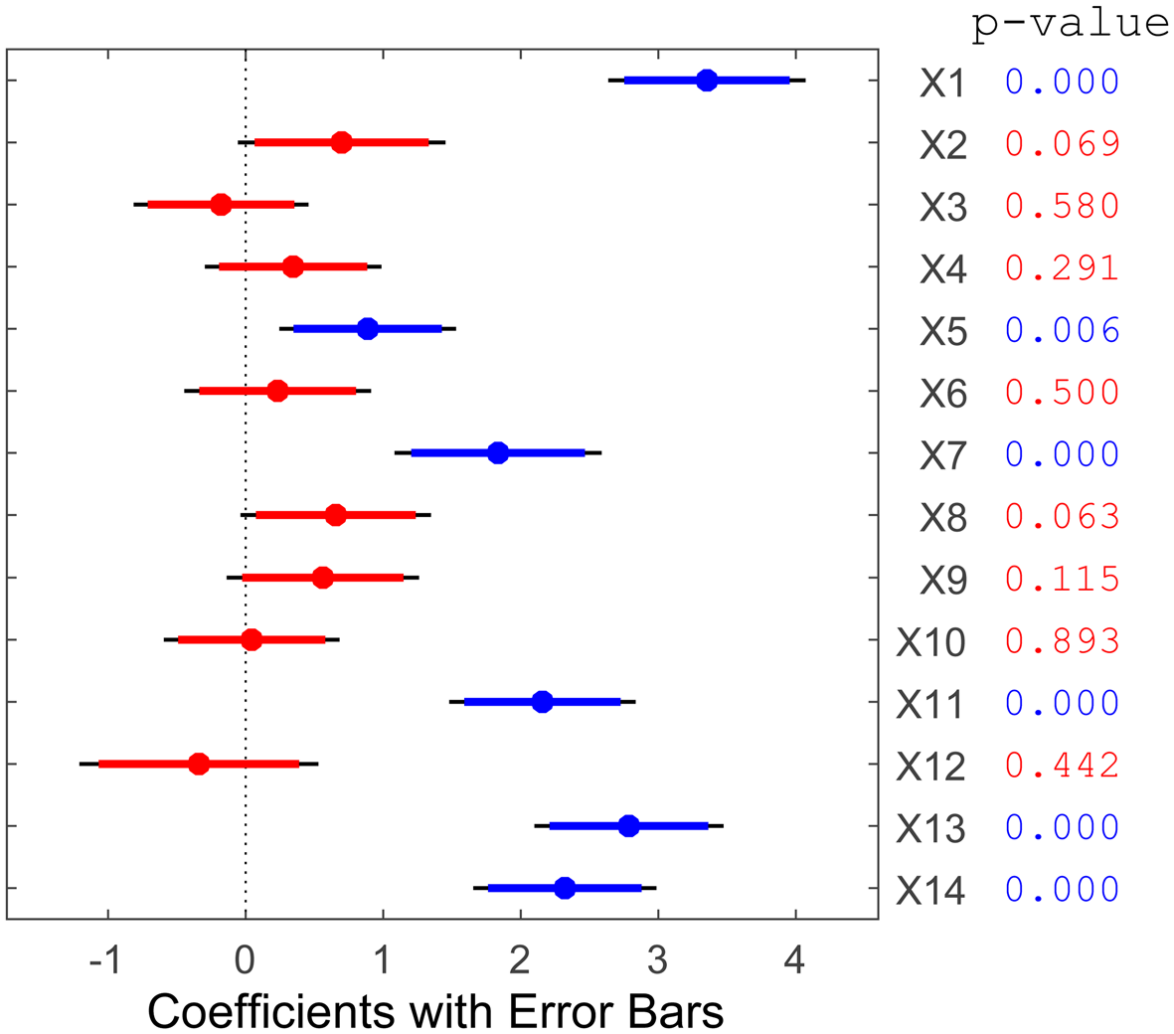
Variance selection using the stepwise regression method.

PCA enables the projection of samples from a high-dimensional space to a lower-dimensional space, revealing the spatial distribution characteristics among different samples in the data^26^. PCA demonstrated a certain degree of overlap in the distribution regions between benign and malignant nodule samples (Fig. 3). This overlap introduces an error rate in diagnosing benign and malignant breast nodules, which is consistent with the diagnosis results of individual ultrasonographic characteristics. Therefore, we could apply several mathematical models to analyze ultrasonographic characteristics and clinical information for diagnosing benign and malignant nodules. The stepwise regression method showed that 6 variances had a significant relationship with the dependent variable, including age (Coeff = 3.553, P < 0.01), background echotexture (Coeff = 0.887, P < 0.01), shape (Coeff = 1.835, P < 0.01), calcification (Coeff = 2.157, P < 0.01), resistance index (Coeff = 2.786, P < 0.01), and axillary lymph node (Coeff = 2.320, P < 0.01) (Fig. 4).

### Model analysis

The model for each mathematical model was built based on the training set using the six variables selected by the stepwise regression method. After 100 random runs of Monte Carlo simulation, the diagnosis results of seven methods in the training and the prediction sets are shown in Table 2. In the training set, the diagnostic rates of all tested methods ranged from 0.849 to 0.999 for benign nodules and from 0.915 to 0.971 for malignant nodules, indicating that the diagnosis rate of all models was satisfactory. In the prediction set, the diagnostic rates of Logistics, PLS-DA, Linear SVM, RF, ANN, KNN and LDA, for benign nodules were 0.845, 0.833, 0.881, 0.850, 0.858, 0.846, and 0.851, respectively, and Linear SVM has the higher values than other methods (P < 0.05). The diagnostic rates of Logistics, ANN and LDA for malignant nodules (ranging from 0.910 to 0.912) were the highest and the diagnostic rate of KNN was the lowest (0.865). Among these methods, only the diagnostic rate of RF and KNN in the prediction sets (ranging from 0.846 to 0.898) were much lower than that in train sets (> 0.947). The AUC value of Linear SVM was the highest (0.890), followed by ANN (0.883), LDA (0.880), Logistics (0.878), RF (0.874), PLS-DA (0.866), and KNN (0.855). The AUC values of all models were higher than individual ultrasonographic characteristics (ranging from 0.494 to 0.764)).

**Table 2:** Diagnosis result of benign and malignant thyroid nodules using different mathematical models.

Method	Training set		Prediction set		ROC
	Benign	Malignant	Benign	Malignant	AUC
	(N=353)	(N=489)	(N=35)	(N=49)	
Logistics	0.872	0.915	0.845	0.912	0.878 (0.871~0.885)*
PLS-DA	0.849	0.918	0.833	0.900	0.866 (0.856~0.877)
Linear SVM	0.883	0.909	0.881	0.899	0.890 (0.882~0.898)
RF	0.947	0.965	0.850	0.898	0.874 (0.864~0.884)
ANN	0.865	0.922	0.858	0.910	0.883 (0.875~0.891)
KNN	0.999	0.971	0.846	0.865	0.855 (0.845~0.866)
LDA	0.851	0.916	0.851	0.910	0.880 (0.872~0.889)

^*^95% confidence intervals in parentheses.

## Discussion

Ultrasound is the primary method used to differentiate between benign and malignant breast nodules^27^. Our result indicated that patient’s age, birth number, shape, resistance index, calcification, axillary lymph node, internal echo, margin, alder grade and background echotexture were significantly correlated with benign and malignant breast nodules. It has been reported that the breast nodules with irregular morphology, indistinct borders, hypoechoic pattern and suspicion of calcifications suggest malignancy^28^. Besides, there existed a significant correlation between nodule size and its pathology. In general, the larger the nodules are, the higher their degree of malignancy. However, it is important to note that nodules with smaller size are often overlooked by patients and sonographers. Neovascularization plays a crucial role in the onset, progression, invasion, and metastasis of breast cancer^29^. The higher the alder grade and resistance index of nodules were, the higher the possibility of malignancy, which should be paid attention to. In the BI-RADS classification, the probability of malignancy ranged from 2 to 95% for nodules in the 4 categories^30^. However, when a nodule is classified as grade 4, breast surgeons may recommend conducting additional examinations for patients, such as mammography, magnetic resonance imaging, or even core needle biopsy. These often increase the economic cost and psychological burden of patients. In addition, BI-RADS classification depends on the experience of physicians^31^. Our results showed that the AUC value of the test models ranged from 0.855 to 0.890, which was better than that of the BI-RADS classification based on junior and senior physicians (0.718-0.790 and 0.766-0.870, respectively) reported in the literature^32,33^. Our finding suggested that all seven models could effectively predict benign and malignant nodules, which could help doctors judge the malignant probability of nodules and reduce unnecessary examinations for patients^34^.

The stepwise regression method showed that age, background echotexture, shape, calcification, resistance index, and axillary lymph node had significant relationships with breast nodule pathology (P < 0.05), suggesting that these variables had a substantial contribution to the model. Using these relevant variables instead of all variables has the potential to enhance model performance, simplify the model, and avoid overfitting^35^. Among these models, Linear SVM had the highest diagnosis rate of benign breast nodules, and Logistics, ANN and LDA had the highest diagnosis rate of malignant breast nodules. Linear SVM could be recommended for diagnosing benign nodules, while Logistics, ANN and LDA could be recommended for diagnosing malignant ones. The diagnosis rate of the KNN and RF in the prediction set was significantly lower than that in the training set, which is likely attributed to model overfitting^36^. Compared with the training set, the results of the prediction set better reflect the performance of the two models. However, the overfitting of RF and KNN in the training set could limit their application in practical work.

Many reports indicate that artificial intelligence technology can classify images directly into benign and malignant categories by extracting feature variables such as color, contour, and texture^37–39^. However, these image feature variables extracted by artificial intelligence technology often lack clinical significance, posing significant limitations during the practical application of the model. In contrast, the clinical information and ultrasonographic characteristics of breast nodules were used for building our models and these variables hold clinical significance. This approach not only uncovers the relationship between breast nodule features and pathology, but also enhances the generalizability of these models.

In our study, these models were constructed using algorithm programs provided by MATLAB software, which could reduce the complexity of data analysis. Indeed, a certain level of programming knowledge is still required when using MATLAB software, particularly for modifying or rectifying inappropriate commands. Additionally, it is important to note that a single computation can result in biased estimates and the Monte Carlo cross-validation method could be employed to obtain robust statistical analysis results.

While mathematical models cannot fully substitute doctors, they can effectively aid in diagnosis. However, there are some limitations to the present study. For example, the present work relied on a limited sample of nodules for assessment. The assessment of controversial nodules by doctors involves subjectivity, and inaccurate judgment of nodule characteristics can further impact the diagnosis^40^. In future work, we will further incorporate more variables, including pathological types and ultrasound elasticity, to improve model diagnosis performance. In addition, we further used ultrasound images coupled with mathematical models to predict the presence of lymph node metastasis in malignant nodules.

## Data Availability

The datasets generated and/or analysed during the current study are not publicly available due to patient privacy protection but are available from the corresponding author on reasonable request.

## References

[1] Kleibl Z, Kristensen VN (2016). Women at high risk of breast cancer: Molecular characteristics, clinical presentation and management. Breast.

[2] Masuda H (2012). Role of epidermal growth factor receptor in breast cancer. Breast Cancer Res. Treat..

[3] Zhang YN, Xia KR, Li CY, Wei BL, Zhang B (2021). Review of breast cancer pathologigcal image processing. Biomed. Res. Int..

[4] Ding R (2022). Breast cancer screening and early diagnosis in Chinese women. Cancer Biol. Med..

[5] Kim SH, Kim HH, Moon WK (2020). Automated breast ultrasound screening for dense breasts. Korean J. Radiol..

[6] Brem RF, Lenihan MJ, Lieberman J, Torrente J (2015). Screening breast ultrasound: Past, present, and future. Am. J. Roentgenol..

[7] Wang J, Chu YH, Wang BH, Jiang TN (2021). A narrative review of ultrasound technologies for the prediction of neoadjuvant chemotherapy response in breast cancer. Cancer Manag. Res..

[8] Linda A (2011). Hyperechoic lesions of the breast: Not always benign. AJR Am. J. Roentgenol..

[9] Arian A, Dinas K, Pratilas GC, Alipour S (2022). The breast imaging-reporting and data system (BI-RADS) made easy. Iran. J. Radiol..

[10] Li JY (2020). Subclassification of BI-RADS 4 magnetic resonance lesions: A systematic review and meta-analysis. J. Comput. Assist. Tomogr..

[11] Hsieh TC, Hsu CW (2019). Breast metastasis from colorectal cancer treated by multimodal therapy case report and literature review. Medicine.

[12] Wang B (2022). Logistic regression analysis of conventional ultrasound, and contrast-enhanced ultrasound characteristics. J. Ultrasound Med..

[13] Salmanpour MR, Rezaeijo SM, Hosseinzadeh M, Rahmim A (2023). Deep versus handcrafted tensor radiomics features: Prediction of survival in head and neck cancer using machine learning and fusion techniques. Diagnostics.

[14] Shi SS, An X, Li YH (2023). Ultrasound radiomics-based logistic regression model to differentiate between benign and malignant breast nodules. J. Ultrasound Med..

[15] Jahangirimehr A (2022). Machine learning approach for automated predicting of COVID-19 severity based on clinical and paraclinical characteristics: Serum levels of zinc, calcium, and vitamin D. Clin. Nutr. ESPEN.

[16] Isik H, Arslan S (2011). An artificial neural network classification approach for use the ultrasound in physiotherapy. J. Med. Syst..

[17] Li YP (2019). Diagnosis of early gastric cancer based on fluorescence hyperspectral imaging technology combined with partial-least-square discriminant analysis and support vector machine. J. Biophoton..

[18] Vejdannik M, Sadr A (2016). Application of linear discriminant analysis to ultrasound signals for automatic microstructural characterization and classification. J. Signal Process. Syst Signal Image Video Technol..

[19] Uchino E, Kubota R, Koga T, Misawa H, Suetake N (2016). Multiple k-nearest neighbor classifier and its application to tissue characterization of coronary plaque. Ieice Trans. Inform. Syst..

[20] Alex DM, Chandy DA, Christinal AH, Singh A, Pushkaran M (2022). A hybrid random forest classifier for chronic kidney disease prediction from 2D ultrasound kidney images. Int. J. Pattern Recogn. Artif. Intell..

[21] Liu JJ (2022). Mammography diagnosis of breast cancer screening through machine learning: A systematic review and meta-analysis. Clin. Experim. Med..

[22] Kim WH, Lee SH, Chang JM, Cho N, Moon WK (2017). Background echotexture classification in breast ultrasound: Inter-observer agreement study. Acta Radiologica.

[23] Chou YH (2001). Stepwise logistic regression analysis of tumor contour features for breast ultrasound diagnosis. Ultrasound Med. Biol..

[24] Du YP, Kasemsumran S, Maruo K, Nakagawa T, Ozaki Y (2006). Ascertainment of the number of samples in the validation set in Monte Carlo cross validation and the selection of model dimension with Monte Carlo cross validation. Chemometr. Intell. Lab. Syst..

[25] Li Y (2013). A novel method to estimate the chemical rank of three-way data for second-order calibration. Chemometr. Intell. Lab. Syst..

[26] Huang YL (2005). Image retrieval with principal component analysis for breast cancer diagnosis on various ultrasonic systems. Ultrasound Obstet. Gynecol..

[27] Gao YH (2020). Clinical value of ultrasound-guided minimally invasive biopsy in the diagnosis or treatment of breast nodules. Cancer Manag. Res..

[28] Hammer MM, Barbosa EJM (2017). Predictive factors for malignancy in incidental pulmonary nodules detected in breast cancer patients at baseline CT. Eur. Radiol..

[29] Zheng Q (2019). Assessment of angiogenesis in rabbit orthotropic liver tumors using three-dimensional dynamic contrast-enhanced ultrasound compared with two-dimensional DCE-US. Jpn. J. Radiol..

[30] Liu YF (2023). High-temporal resolution DCE-MRI improves assessment of intra- and peri-breast lesions categorized as BI-RADS 4. BMC Med. Imaging.

[31] Wojcinski S, Stefanidou N, Hillemanns P, Degenhardt F (2013). The biology of malignant breast tumors has an impact on the presentation in ultrasound: An analysis of 315 cases. BMC Womens Health.

[32] Liu MH, He F, Xiao JD (2022). Application of S-detect combined with virtual touch imaging quantification in ultrasound for diagnosis of breast mass. J. Central South Univ. Med. Sci..

[33] Xia Q (2021). Differential diagnosis of breast cancer assisted by S-Detect artificial intelligence system. Math. Biosci. Eng..

[34] Schnitt SJ (2019). Problematic issues in breast core needle biopsies. Modern Pathol..

[35] Wang MC, Wright J, Brownlee A, Buswell R (2016). A comparison of approaches to stepwise regression on variables sensitivities in building simulation and analysis. Energy Build..

[36] Cosenza DN (2021). Comparison of linear regression, k-nearest neighbour and random forest methods in airborne laser-scanning-based prediction of growing stock. Forestry.

[37] Fraiwan M, Faouri E, Khasawneh N (2022). Multiclass classification of grape diseases using deep artificial intelligence. Agriculture-Basel.

[38] Salmanpour MR, Hosseinzadeh M, Rezaeijo SM, Rahmim A (2023). Fusion-based tensor radiomics using reproducible features: Application to survival prediction in head and neck cancer. Comput. Methods Programs Biomed..

[39] Rezaeijo SM, Nesheli SJ, Serj MF, Birgani MJT (2022). Segmentation of the prostate, its zones, anterior fibromuscular stroma, and urethra on the MRIs and multimodality image fusion using U-Net model. Quant. Imaging Med. Surg..

[40] Rezaeijo SM, Chegeni N, Naeini FB, Makris D, Bakas S (2023). Within-modality synthesis and novel radiomic evaluation of brain MRI scans. Cancers.

